# Purification of the full-length, membrane-associated form of the antiviral enzyme viperin utilizing nanodiscs

**DOI:** 10.1038/s41598-022-16233-z

**Published:** 2022-07-13

**Authors:** Ayesha M. Patel, Karl J. Koebke, Timothy J. Grunkemeyer, Colleen M. Riordan, Youngsoo Kim, Ryan C. Bailey, E. Neil G. Marsh

**Affiliations:** 1 Department of Chemistry, University of Michigan, Ann Arbor, MI 48109 USA; 2 Department of Biological Chemistry, University of Michigan, Ann Arbor, MI 48109 USA

**Keywords:** Biochemistry, Biological techniques, Biophysics

## Abstract

Viperin is a radical S-adenosylmethionine enzyme that catalyzes the formation of the antiviral ribonucleotide, 3’-deoxy-3’,4’-didehydroCTP. The enzyme is conserved across all kingdoms of life, and in higher animals viperin is localized to the ER-membrane and lipid droplets through an N-terminal extension that forms an amphipathic helix. Evidence suggests that the N-terminal extension plays an important role in viperin’s interactions with other membrane proteins. These interactions serve to modulate the activity of various other enzymes that are important for viral replication and constitute another facet of viperin’s antiviral properties, distinct from its catalytic activity. However, the full-length form of the enzyme, which has proved refractory to expression in *E. coli*, has not been previously purified. Here we report the purification of the full-length form of viperin from HEK293T cells transfected with viperin. The purification method utilizes nanodiscs to maintain the protein in its membrane-bound state. Unexpectedly, the enzyme exhibits significantly lower catalytic activity once purified, suggesting that interactions with other ER-membrane components may be important to maintain viperin’s activity.

## Introduction

Viperin (Virus Inhibitory Protein, Endoplasmic Reticulum-associated Interferon iNducible)^1–3^ is a radical S-adenosylmethionine (SAM) enzyme that was recently shown to catalyze the radical-mediated dehydration of CTP to produce the modified nucleotide 3’-deoxy-3’,4’-didehydroCTP (ddhCTP)^4^. ddhCTP acts as a chain-terminating inhibitor of many virally encoded RNA-dependent RNA polymerases^4,5^. Viperin-like enzymes appear conserved in all six kingdoms of life, pointing to an ancient role for this family enzymes in combating viral infections^5–7^.

In higher animals, viperin expression is highly up regulated by type I interferons and viruses^2,8,9^. The enzyme is integrated into the broader innate immune response to viral infection and has been shown to regulate both the activity and expression levels of numerous cellular and viral proteins. This network of protein–protein interactions results in the enzyme exerting a wide array of antiviral properties beyond just the synthesis of ddhCTP. Cellular enzymes shown to interact with viperin include the mitochondrial trifunctional protein^10,11^, which is involved in fatty acid β-oxidation; squalene monooxygenase and lanosterol synthase^12,13^, which catalyze key steps in sterol biosynthesis; and interleukin receptor-associated kinase 1 (IRAK1) and the E3 ubiquitin ligase, TRAF6^14–16^, which are components of the TLR7/9 innate immune signaling pathway. Viperin also binds to a wide range of viral proteins from viruses such as hepatitis C, Dengue, tick-borne encephalitis and Zika viruses^2,8,9^. Depending on the virus, viperin interacts with both non-structural proteins that are responsible for viral replication and assembly and with structural proteins that form the viral capsid^17–19^.

In animals, viperin is localized to the cytosolic face of the ER membrane and lipid droplets through an N-terminal extension that is unstructured in solution but adopts an amphipathic α-helical conformation upon binding the lipid bilayer^20^. Although the membrane-localizing sequence is not required for enzymatic activity, the N-terminal extension likely plays an important role in mediating viperin’s interactions with other proteins—either by making direct contacts with other proteins or, indirectly, by localizing viperin to the ER membrane and lipid droplets. For example, we recently showed by co-immunoprecipitation that viperin forms a complex with the hepatitis-C viral protein, NS5B, and the cellular protein, VAP33, both of which also bind to the ER membrane^19^. However, these interactions were lost when the membrane-localizing sequences were removed to facilitate expression and purification of these proteins from *E. coli*.

So far, it has not proved possible to express full-length viperin (i.e. the protein that includes the N-terminal ER-membrane localizing sequence) as recombinant protein in a bacterial expression system such as *E. coli*. Therefore, most mechanistic studies and all structural studies on the enzyme have been conducted on truncated constructs lacking this sequence, which can be over-expressed and purified from *E. coli*^4,6,7^. Here, we report the purification of the full-length enzyme from transfected HEK293T cells in which we have used nanodiscs to retain the interaction between the N-terminal sequence and the lipid bilayer. Nanodiscs are discoidal lipid bilayers, ranging in diameter between 8 and 16 nm^21,22^. The discoidal structure is stabilized by alpha-helical proteins known as membrane scaffold proteins (MSPs) that are derived from apolipoprotein A1 and form a ‘belt’ around the disc that stabilizes lipid bilayers in solution^23^. This work represents the first purification of functional full-length viperin from a mammalian system. The ability to purify the full-length, membrane associated form of viperin should facilitate efforts to elucidate the molecular mechanisms by which viperin interacts with other membrane-associated protein and the effect of these interactions on viperin’s antiviral activity.

## Materials and methods

### Cell lines

The HEK293T cell line was purchased from ATCC.

### Plasmids

A synthetic gene encoding human viperin (GenBank accession no. AAL50053.1) was synthesized and subcloned into pcDNA3.1(+) vector (GenScript). A 3X FLAG tag (DYKDHDGDYKDHDIDYKDDDDK) was introduced at the N-terminus of the protein to facilitate purification. A Kozak consensus sequence (5′-GCCAAC-3′) was introduced ahead of the gene to facilitate expression in eukaryotic cells. The gene encoding the membrane scaffold protein with an E3 amphipathic helical region insertion (MSP1E3D1) was cloned into pET28a(+) vector and equipped with an N-terminal His tag to facilitate purification.

### Reagents and antibodies

For DNA transfection in HEK293T cells, transfection grade linear polyethylenimine (PEI) hydrochloride was purchased from Polysciences, Inc. *S*-(5′-Adenosyl)-L-methionine p-toluenesulfonate salt and cytidine 5′-triphosphate disodium salt hydrate were purchased from Sigma Aldrich. POPC (1-palmitoyl-2-oleoyl-sn-glycero-3-phosphocholine,) and POPE (1-palmitoyl-2-oleoyl-sn-glycero-3-phosphoethanolamine) were purchased from Avanti Polar Lipids. Amberlite XAD-2 hydrophobic beads, Anti-FLAG M2 magnetic beads, and 3X FLAG peptide were purchased from Millipore Sigma. Pierce silver stain kit was purchased from ThermoFisher Scientific. Rabbit polyclonal viperin antibody (11833–1-AP) was purchased from ProteinTech. Goat anti-rabbit Ig secondary antibody (170-6515) was purchased from BioRad.

### Cell culture and transfection

The gene encoding viperin housed in the pcDNA3.1 shuttle vector was overexpressed in HEK293T cells (cultivated in DMEM supplemented with 10% FBS and 1% penicillin–streptomycin) using standard transient transfection protocols with polyethylenimine transfecting agent, as described previously^24^. Typically, 50 µg of DNA was mixed with 100 µg PEI in 1:2 ratio (w/w) and incubated for 10 min at room temperature before adding it to HEK293T cells at 50–60% confluence in a 150 mm culture dish. The cells were grown at 37 °C under CO_2_ for 36–40 h, gently harvested, and cell pellets were stored at − 80 °C.

### Membrane scaffold protein (MSP) expression and purification

MSP1E3D1 (MSP) containing an N-terminal His tag was expressed in *E. coli* BL21 (DE3) and purified by Ni–NTA metal affinity chromatography as described previously^25^. Further purification by ion exchange chromatography^26^ was found to be necessary to remove protease contamination remaining in the MSP solution. A prepacked Q-Sepharose (GE healthcare) column was equilibrated in 40 mM Tris–HCl pH 7.4, 100 mM NaCl buffer. Ni-purified MSP was loaded onto the column and proteins were eluted with an increasing concentration of NaCl. MSP eluted at ~ 200 mM NaCl. The purified protein was buffer exchanged into 40 mM Tris–HCl pH 7.4, 100 mM NaCl, concentrated, and stored at − 80 °C.

### Preparation of lipid solutions for nanodiscs

The nanodisc lipid composition for viperin incorporation was optimized at 80% POPC and 20% POPE. POPC and POPE were dissolved in chloroform and mixed in the desired ratio and dried under nitrogen. The dried lipids were stored in a desiccator under vacuum overnight. For nanodisc assembly, a stock solution (25 mM) was prepared by dissolving the dried lipid mixture in buffer A (50 mM HEPES pH 7.4, 150 mM NaCl) with 100 mM Na-cholate by sonication in a water bath for 30 min until the solution turned clear. The lipid solution was mixed with MSP1E3D1 in 20 mM Na-cholate and HEPES buffer in a final molar ratio of 130:1 (phospholipid:MSP).

### Viperin incorporation into nanodiscs: assembly and purification

All steps were performed inside a Coy anaerobic chamber unless mentioned otherwise. All buffers and stock solutions were thoroughly degassed before introducing them into the anaerobic chamber. Incubations at 4 °C were performed outside the chamber; the tubes were tightly wrapped with parafilm to exclude air before being removed from the chamber.

HEK293T cells transfected with viperin were harvested from four 150 mm culture dishes and resuspended in 500 µL (total) of 1X TBS (20 mM Tris–HCl pH 7.4, 150 mM NaCl) with 5 mM DTT, 0.1% (w/v) Tween-20 and protease inhibitor cocktail and sonicated for 10 pulses. Each plate yielded ~ 2 × 10^7^ cells. The cell lysate was cleared by centrifugation at 12,500 rpm for 20 min at 4 °C. A portion of lysate was saved for assay of viperin activity. The total protein concentration in the cell lysate was then measured using the BCA assay. In an Eppendorf tube, the cell lysate was then incubated with the phospholipid/MSP solution at 4 °C for 2 h with end-to-end mixing. The amount of phospholipid/MSP was adjusted so that the concentration of MSP was approximately double that of the proteins in the cell lysate. In a typical purification the cell lysate protein concentration was ~ 16 µM and the concentrations of phospholipid and MSP were 4.2 mM and 32 µM respectively in a total volume of 2.5 mL. To remove the detergent and facilitate nanodisc assembly, Amberlite XAD-2 beads were then added in equal volume (2.5 mL) to the lysate, MSP, and lipid mixture and incubated at 4 °C overnight.

### Ni–NTA metal affinity chromatography

The nanodisc-lysate mixture was filtered through 0.22 µm syringe filter to remove the Amberlite beads. Ni–NTA beads (~ 1 mL) were equilibrated with buffer B (50 mM HEPES pH 7.4, 150 mM NaCl, 30 mM imidazole) and incubated with nanodisc lysate mixture at 4 °C for 1 h. The resin was then washed with 4 column volumes of buffer B twice before eluting protein with 2 column volumes of buffer C (50 mM HEPES pH 7.4, 150 mM NaCl, 300 mM imidazole). The eluted protein was concentrated by ultrafiltration against a 10 kDa cutoff membrane. A portion of concentrated Nickel-NTA-purified nanodisc sample was saved for assay of viperin activity.

### Anti-FLAG affinity chromatography

The concentrated, nickel-affinity purified nanodisc sample (~ 900 µL) was incubated with anti-FLAG magnetic beads (~ 40 µL) equilibrated with buffer A (50 mM HEPES pH 7.4, 150 mM NaCl) at 4 °C for 16 h. The resin was washed with 4 column volumes of buffer A three times. The viperin incorporated nanodiscs were finally eluted by incubating anti-FLAG magnetic beads with 3X FLAG peptide solution (2.5 µg/µL) prepared in buffer A at 4 °C for 2 h with end-to-end mixing. The purity of the protein was analyzed by SDS-PAGE (4–20% gradient gel), with proteins visualized by silver staining. To confirm the presence of viperin, the duplicate SDS-PAGE gels were immunoblotted against anti-viperin antibody using standard protocols.

### Reconstitution of the iron-sulfur cluster of viperin

The NTA- and FLAG-purified viperin nanodiscs samples were incubated with 5 mM DTT at 4 °C for 20 min. Stock solutions of FeCl_3_ (100 µM) and Na_2_S (100 µM) were then added slowly in small additions (5 additions of 2 µL each, reaching to final concentration of 10 µM with 30 min incubation after each addition) to fully reconstitute the [4Fe-4S] cluster of viperin, as described previously^24^.

### Viperin assay

Viperin activity was assayed under anaerobic conditions in a Coy anaerobic chamber as follows: Samples of HEK293T cell lysate, nickel-affinity purified nanodiscs, or FLAG-affinity purified viperin nanodiscs (100 µL each) were initially incubated with 5 mM dithionite and 0.3 mM CTP (final concentrations) at 25 °C for 20 min. The reaction was initiated by the addition of 0.3 mM SAM (final concentration) to the sample and incubated at 25 °C for 1 h with end-to-end mixing. The concentrations of CTP and SAM were assumed to be saturating based on the previously reported kinetic parameters^4,6^ for the N-terminal truncated forms of the enzyme that have been expressed and purified from *E. coli*.

The reaction mixtures were then removed from the Coy chamber and quenched by heating at 95 °C for 10 min followed by incubation on ice for 5 min. The precipitated proteins were removed by centrifugation at 14,000 rpm for 20 min. 5′-dA was extracted from the supernatant (~ 90 µL) with a fivefold excess of acetonitrile (450 µL). Samples were analyzed in triplicate by UPLC-tandem mass spectrometry as described previously^12^.

The amount of viperin in the various samples was determined by immunoblotting with reference to a standard curve constructed from purified, truncated enzyme as described previously^15^. The activity of the various samples was normalized with respect to the amount of viperin in the sample.

### Imaging of gels and blots

Silver stained polyacrylamide gels and immuno-stained western blots were imaged using a BioRad ChemiDoc™ Touch system. Imaging of immuno-stained blots was performed using the instrument’s standard chemiluminescent application with autoexposure; silver stained gels were imaged using white light transillumination with autoexposure.

### Electron microscopy of nanodiscs

Protein samples were negatively stained using uranyl formate on FCF100-Cu grids (Electron Microscopy Sciences) for imaging. Grids were imaged on a Morgagni electron microscope with a high tension of 100 kV, at 22,000 × magnification, and a pixel size of 2.1 Å/pixel at this magnification.

## Results and discussion

### Purification of viperin into nanodiscs

Mammalian membrane-associated proteins such as viperin have proven to be especially challenging to recombinantly express and purify from bacteria such as *E. coli*. This is because, lacking the requisite membrane structure to bind and chaperones that may be needed for them to fold, they are prone to misfolding and are rapidly degraded by the host cell. Thus, despite multiple attempts using various *E. coli* strains developed to facilitate the expression of “difficult to express” proteins we were unable to detect expression of full-length viperin. In contrast, we have found that the N-terminal truncated form of the enzyme expresses well in *E. coli*^14,19^. However, previous studies in our laboratory had established that viperin is expressed well in HEK293T cells transfected with the pcDNA3.1 (+) expression vector housing a gene for the full-length enzyme^12,15,24^. Therefore, we sought to develop a method to purify the full-length enzyme from transfected HEK293T cells.

**Figure 1:**
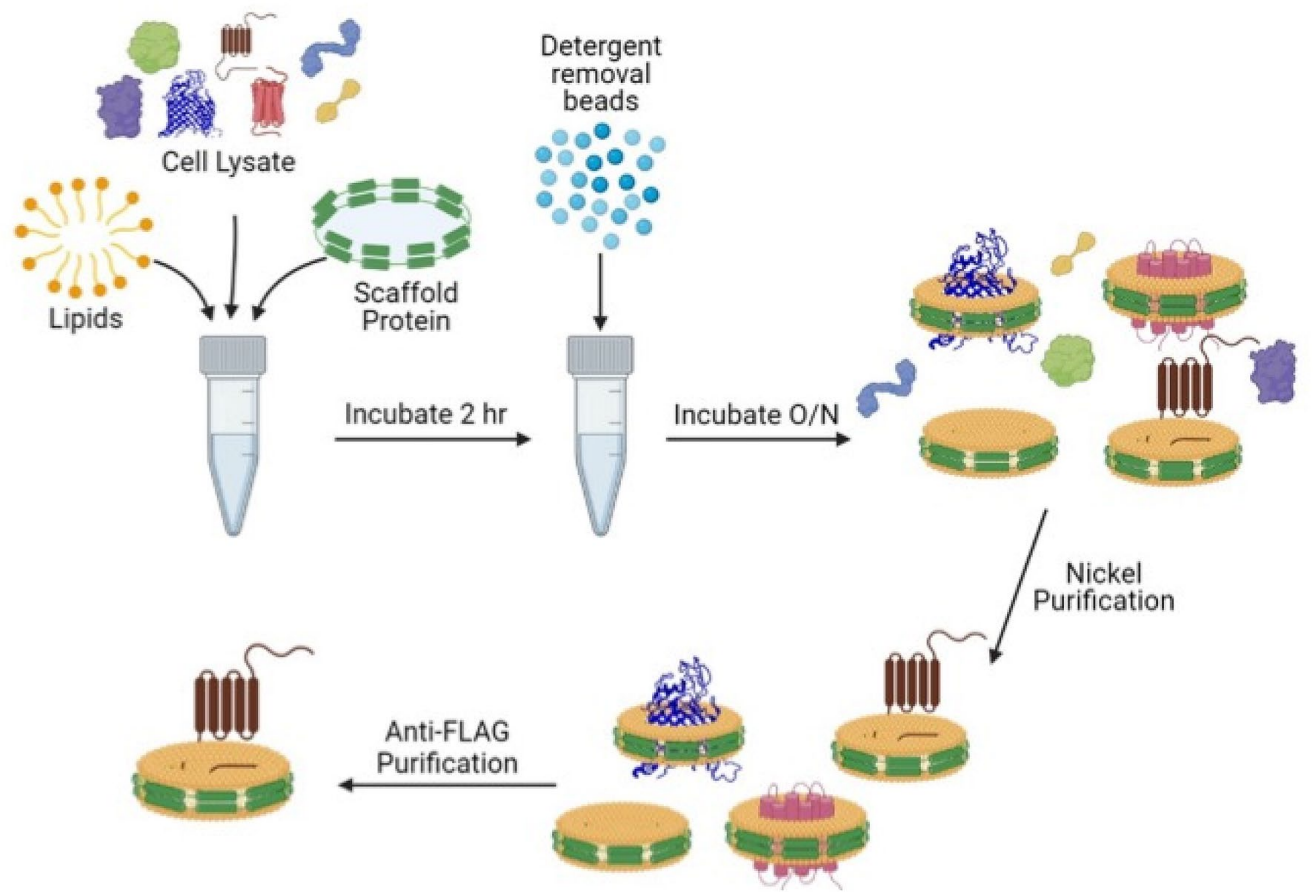
Scheme showing an overview of the strategy for purifying full-length viperin incorporated into nanodiscs. Lysate from HEK293T cells transiently transfected with viperin is mixed with scaffold protein and lipids in the presence of detergent. Removing the detergent results in the spontaneous formation of nanodiscs. Next, the nanodiscs are purified using the His-tag on the scaffold protein. Lastly, viperin-containing nanodiscs are further purified using the 3xFLAG tag on viperin.

Initial attempts to purify viperin directly from HEK293T cells utilizing a 3X FLAG-tag introduced to at the N-terminus of viperin (either with or without non-ionic detergents present) were unsuccessful and resulted in very low yields of inactive enzyme. Therefore, to facilitate purification of viperin, which localizes to the ER-membrane through its N-terminal amphipathic helix^20^, we employed lipid nanodiscs to provide a native-like environment that would substitute for the ER-membrane^21^. The purification strategy is illustrated in Fig. 1: first membrane proteins in clarified cell lysates are extracted into nanodiscs; then the nanodiscs are purified from the lysate using the His-tag appended to the MSP; lastly, viperin-containing nanodiscs are purified from the mixture by using a 3X FLAG-tag introduced to at the N-terminus of viperin.

To obtain sufficient material to purify viperin, 3 or 4 150-mm culture dishes were used to culture viperin-transfected HEK293T cells, with each dish yielding approximately 20 × 10^6^ cells at confluency. Cells were either used immediately after harvesting or flash frozen and stored at – 80 °C. Because viperin, like all radical SAM enzymes contains an oxygen-sensitive [4Fe-4S] cluster^27^, all lysis and purification steps were performed in an anaerobic glove box.

After lysis by sonication and centrifugation to remove cellular debris, the clarified lysate was incubated with the scaffold protein, MSP1E3D1, a mixture of 80% POPC and 20% POPE lipids (preliminary experiments established that this ratio appeared to give the highest incorporation of viperin into nanodiscs) and sodium cholate to facilitate extraction of membrane proteins into nanodiscs. MSP1E3D1 is designed to facilitate the formation of nanodiscs of ~ 12.9 nm diameter^25^ which is appropriate to incorporate one molecule of viperin per nanodisc. To maximize the incorporation of viperin, the final concentration of MSP1E3D1 was adjusted to be approximately double the nominal concentration of cellular proteins in the lysate, assuming an average molecular weight of 350 kDa for a hypothetical human cellular protein. Based on this number, the molar ratio of lipids (POPC + POPE) to MSP1E3D1 was then adjusted to be 135:1. In a typical experiment, the final concentrations of each of the components was 16.2 µM nominal concentration of cell lysate proteins (equivalent to 5.67 mg/mL), 32.4 µM MSP1E3D1, 4.2 mM POPC + POPE, and 20 mM Na cholate. The mixture was incubated in buffer A (50 mM HEPES pH 7.4, 150 mM NaCl) at 4 °C for 2 h with gentle mixing, after which Amberlite XAD-2 beads were added to remove the detergent and incubation was continued at 4 °C overnight. Removing the detergent resulted in spontaneous assembly of the nanodiscs.

**Figure 2:**
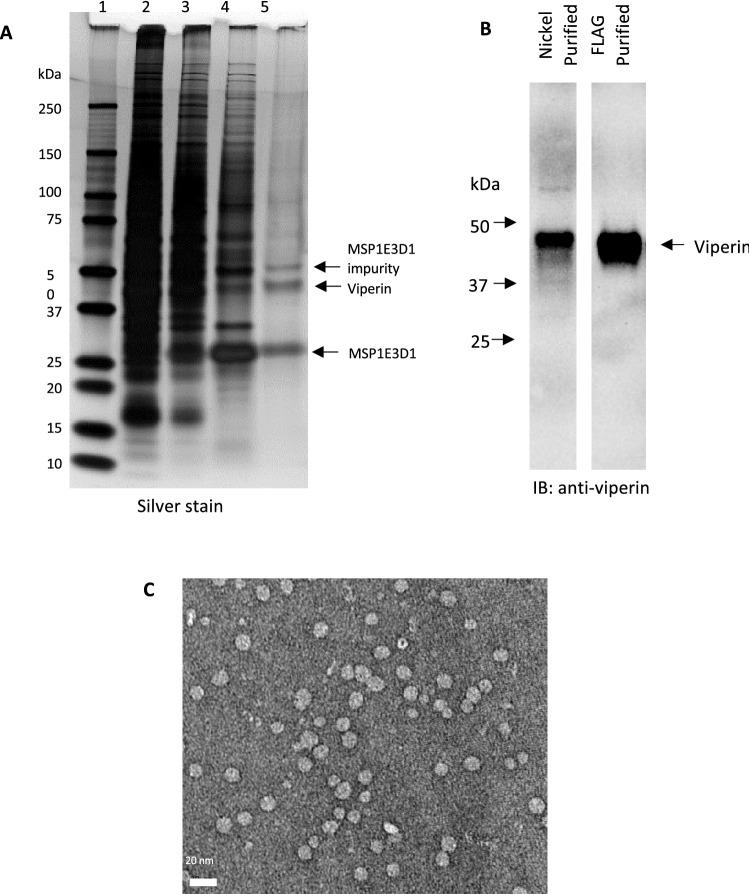
(**A**) SDS PAGE (silver-stained gel) illustrating a typical purification of viperin: *lane 1*: molecular weight ladder; *lane 2*: total cellular protein; *lane 3*: clarified cell lysate prior to addition of MSP; *lane 4*: nanodiscs purified by nickel-NTA affinity chromatography; *lane 5*: viperin nanodiscs purified by FLAG affinity chromatography. (**B**) Immunoblot analysis (blot immunostained for viperin) of the nanodiscs after purification by nickel-NTA affinity and FLAG affinity chromatography confirms the identity of the enzyme. (The uncropped image for panel B is shown in Figure S1) (**C**) Negative stain TEM image of the viperin nanodiscs after purification by FLAG affinity chromatography.

After incorporating proteins into the nanodiscs, the nanodiscs were purified from the cell lysate by Ni–NTA affinity chromatography using the His-tag appended to MSP1E3D1. This step yielded nanodiscs containing viperin and other membrane-bound proteins along with free MSP1E3D1 (Fig. 2A). To purify the nanodiscs containing viperin from nanodiscs containing other proteins, a second affinity purification utilizing the FLAG-tag appended to the N-terminus of viperin was performed. The binding and release of protein onto the anti-FLAG beads is slow, therefore, the nanodiscs were incubated overnight at 4 °C with constant agitation. The beads were then thoroughly washed with buffer A (50 mM HEPES pH 7.4, 150 mM NaCl) to remove unbound nanodiscs and the viperin-containing nanodiscs, were eluted from the beads with high concentrations of 3X FLAG peptide (2.5 mg/mL). However, even after several hours of incubation, only ~ 50% of the viperin nanodiscs were eluted, considerably lowering the yield of purified protein. Attempts to streamline the purification procedure by omitting the Ni–NTA affinity chromatography and purifying viperin nanodiscs directly from the cell lysate using the 3X FLAG tag on viperin were unsuccessful. The small amounts of viperin obtained this way were significantly contaminated with other proteins.

The purity of the viperin nanodiscs was examined using SDS-PAGE (Fig. 2A). The silver-stained gel showed two prominent bands—one at ~ 30 kDa corresponding to MSP1E3D1, and one at ~ 42 kDa representing full-length viperin. (The faint band around ~ 50 kDa corresponds to an impurity in MSP1E3D1 as it was also seen in empty nanodisc samples.) The identity of viperin was further confirmed by immunostaining (Fig. 2B, Figure S1). The relative intensities of the bands due to viperin and MSP1E3D1 are similar, and are consistent with an average of one molecule of viperin being incorporated into a nanodisc formed from two molecules of MSP1E3D1. The integrity of the nanodiscs was examined by transmission electron microscopy (TEM). The images (Fig. 2C) confirmed that nanodiscs of the expected size, 10–15 nm, were present, although as expected the negative stain technique did not allow individual viperin molecules within the nanodiscs to be visualized.

### Activity of purified full-length viperin

We next examined how the specific activity of viperin varied at each step of the purification. The amount of viperin present at each step was determined by immunoblotting, as described previously^15^, by using a standard curve constructed from known amounts of N-terminal-truncated viperin, which was expressed and purified from *E. coli*. After reconstituting the [4Fe-4S] cluster, the activity of viperin was assayed by measuring the formation of 5’-dA when the dithionite-reduced enzyme was incubated with SAM and CTP, as described previously^19^. These data are summarized in Table 1.

**Table 1:** Summary of yield and specific activity (calculated as a turnover number) for the purification of viperin from HEK293T lysate utilizing nanodiscs.

Purification step	Amount of viperin (µg)	Yield (%)	Specific activity (h^−1^)
Cell lysate	4.8	100	3.8 ± 0.6
Nickel-purified nanodiscs	2.7	56	3.8 ± 0.8
FLAG-purified nanodiscs	0.7	15	0.5 ± 0.09

The amount of viperin was quantified by immunoblotting as described in the text. The amount of viperin and percentage yield are values obtained from one representative purification. The specific activity is calculated as an average determined from three purifications.

In the crude HEK293T cell lysate, the specific activity of viperin, calculated as an apparent turnover number, was *k*_obs_ = 3.8 ± 0.6 h^−1^ which is comparable to previously reported values^15^. The specific activity of viperin was essentially unchanged after extraction into nanodiscs and purification by NTA-affinity chromatography. However, somewhat surprisingly, the final purification step significantly reduced the specific activity of viperin by ~ 8-fold to *k*_obs_ = 0.5 ± 0.09 h^−1^. This reduction in activity was observed over several independent purifications.

It is unclear why the activity of the purified enzyme is reduced so dramatically. Incorporating the enzyme into nanodiscs per se does not seem to affect the activity. One possibility is that nonspecific interactions between viperin and the anti-FLAG beads used in the purification result in partial misfolding of the protein. This explanation may be in accord with the poor recovery of enzyme eluted from the anti-FLAG beads, discussed below, which is a problem often encountered using this technique. A more interesting possibility, however, is that other protein(s) or small molecules present in the cell lysate are involved in stabilizing or activating viperin. Such a protein or small molecule would presumably be associated with the ER membrane or liposomes because it would have to co-purify with the nanodiscs isolated by NTA-affinity chromatography. In support of this idea, we note that we have observed in other studies that the activity of viperin can be altered quite significantly by its interactions with other proteins. For example, co-expression of viperin with IRAK1 and TRAF6 in HEK293T cells results in these enzymes forming a complex, with the activity of viperin increasing ~ tenfold as a result^15^. Therefore it seems reasonable that interactions with other endogenous proteins or other components of the lipid membrane may help stabilize the enzyme.

The yield of viperin after the final purification step was calculated to be ~ 15% (~ 0.7 µg, 0.175 µg/150 mm culture dish). Notably, a large loss of protein accompanied the final step in the purification. This is due in part to difficulties encountered in eluting the enzyme from the anti-FLAG beads using the 3x-FLAG peptide. Even after prolonged incubation only about 50% of the protein bound to the beads could be eluted. After taking these losses into account, the amount of purified full-length viperin obtained is comparable to other membrane proteins purified with the aid of nanodiscs: for example, ~ 0.25 µg/100 mm culture dish of the family B GPCR (GLP1R) receptor protein was obtained when purified from a mammalian cell line using a similar approach^28^.

## Conclusions

Here we describe, for the first time, the purification of full-length human viperin, rather than its truncated form, from a transfected mammalian cell line. Purification has been facilitated by the use of nanodiscs that allow the interaction between the amphipathic N-terminal extension of viperin and the lipid bilayer to be preserved. Surprisingly, the purified enzyme has rather low activity. It is currently unclear whether the loss of activity results from the purification conditions employed, which might result in some misfolded protein, or whether it reflects the removal of an unknown activating factor that is presumably retained in the nanodiscs. We consider the latter explanation more likely, given that viperin is known to interact with multiple cellular proteins that alter its specific activity. If correct, our observations imply that the activating factor(s) is incorporated into a different nanodisc from viperin and that the interactions between them are sufficiently weak that the factor does not co-purify with viperin. However, further experiments are needed to better understand what additional factors contribute to viperin’s enzymatic activity.

Although the amount of protein that can be purified from HEK293T cells by this method is relatively small, the amounts are nevertheless sufficient to undertake further studies on the factors affecting viperin’s catalytic activity and to undertake structural studies using single-molecule techniques such as cryo-EM. Such studies could reveal the details of the interaction between viperin and the membrane that appear to be important for its biological function. Furthermore, the ability to purify viperin bound to nanodiscs should facilitate structural characterization of the various complexes that viperin forms with other proteins that make important contributions to its multifaceted antiviral activity.

## Supplementary Information

Supplementary Information.

## Data Availability

The protein sequence for viperin (Radical S-adenosyl methionine domain-containing protein 2): is deposited in the UniProtKB data base: accession code Q8WXG1. The crystal structure of viperin lacking the N-terminal membrane-binding domains is deposited in the Protein Data Base, PDB ID 5VSL. The original uncropped image of the immunoblot shown in Fig. 2 is included as supporting information.

## Abbreviations

ddhCTP: 3’-Deoxy-3’, 4’-didehydro-CTP
Viperin: Virus inhibitory protein, endoplasmic reticulum-associated, interferon-inducible

## Code availability

Viperin (Radical S-adenosyl methionine domain-containing protein 2): Q8WXG1 (UniProtKB), 5VSL (PDB).
