# Supplementary material for: Purification of the full-length, membrane-associated form of the antiviral enzyme viperin utilizing nanodiscs
Source: Sci Rep. 2022 Jul 13;12:11909. doi: 10.1038/s41598-022-16233-z (PMC9279394; doi:10.1038/s41598-022-16233-z)
Supplement: Supplementary file 1 — Supplementary Information. [file 41598_2022_16233_MOESM1_ESM.pdf]

SUPPORTING INFORMATION

Purification of the Full-length, Membrane-associated form of the Antiviral Enzyme Viperin Utilizing Nanodiscs

Ayesha M. Patel[1], Karl J. Koebke[1], Timothy J. Grunkemeyer[1], Colleen M. Riordan [1], Youngsoo Kim[1], Ryan C. Bailey[1] & E. Neil G. Marsh[1,2,]*

[1]Department of Chemistry and [2]Department of Biological Chemistry, University of Michigan, Ann Arbor, MI-48109

Supporting information:

Complete image of the immunoblot stained with anti-viperin antibody used to construct Figure 2b, indicating regions reproduced in Figure 2B.

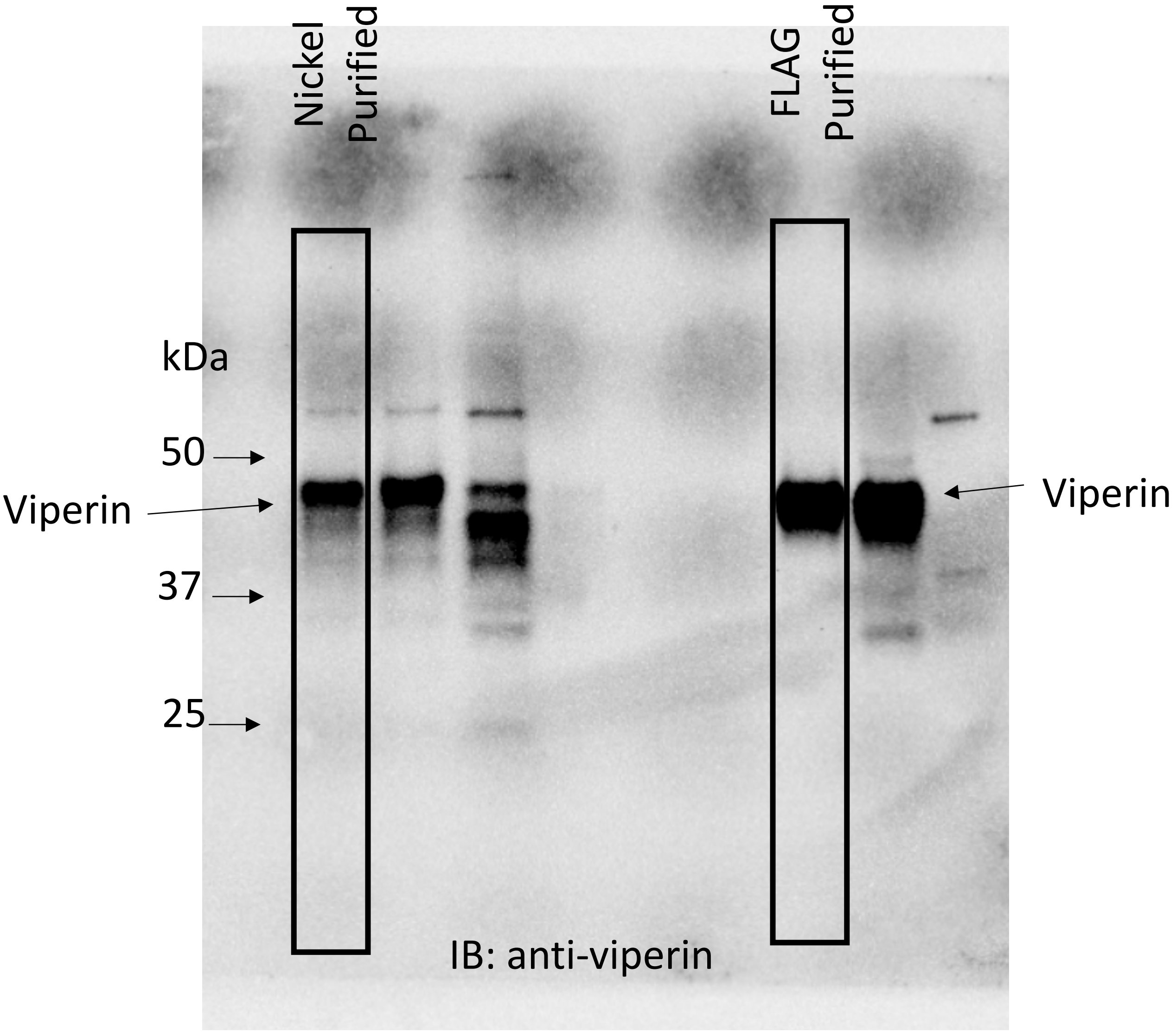

Figure S1: Complete image of the immunoblot stained with anti-viperin antibody used to construct Figure 2B. The other samples shown are unrelated to the experiment described. The regions reproduced in Figure 2B are indicated by boxes.
